# Satellite DNA Amplification in Advanced Prostate Cancer Is Largely Independent From Euchromatic and Oncogene Amplicons

**DOI:** 10.1369/00221554251323657

**Published:** 2025-03-17

**Authors:** Anja Weise, Antonio Augusto Ornellas, Gilda Alves, Constanze Pentzold, Jenny Holler, Melanie Wolter, Elena Jamali, Bernhard Theis, Thomas Liehr

**Affiliations:** Institute of Human Genetics, Jena University Hospital, Friedrich Schiller University, Jena, Germany; Urology Service, Hospital Mário Kröeff, Rio de Janeiro, Brazil; Circulating Biomarkers Laboratory, Faculty of Medical Sciences, Department of General Pathology, Rio de Janeiro State University, Rio de Janeiro, Brazil; Institute of Human Genetics, Jena University Hospital, Friedrich Schiller University, Jena, Germany; Institute of Human Genetics, Jena University Hospital, Friedrich Schiller University, Jena, Germany; Institute of Human Genetics, Jena University Hospital, Friedrich Schiller University, Jena, Germany; Institute of Human Genetics, Jena University Hospital, Friedrich Schiller University, Jena, Germany; Section Pathology, Institute of Forensic Medicine, Jena University Hospital, Friedrich Schiller University, Jena, Germany; Institute of Human Genetics, Jena University Hospital, Friedrich Schiller University, Jena, Germany

**Keywords:** chromosome microarray (CMA), fluorescence in situ hybridization (FISH), heterochromatin, prostate cancer (PCa), satellite DNA

## Abstract

Recently, we were able to show that satellite DNA amplification (satDNA-AMP) is present in advanced prostate cancer. A chromosome microarray study provided first evidence that satDNA-AMP appears to be largely independent of centromere-near/pericentric euchromatic copy number alterations. Therefore, it might be carefully suggested that satDNA-AMP could be a new and independent marker for advanced tumor progression:

## Introduction

Recent studies by ourselves^1^ and others^2^ have shown that satellite DNA is not only expressed as RNA, but is also amplified in advanced prostate cancer (PCa). As described in the study by Ariffen et al.,^1^ amplification of satellite DNA (satDNA-AMP) has already been observed in breast carcinomas, liposarcomas, retinoblastomas, melanomas, and gliomas. The satDNA-AMPs are mostly visible as homogeneously staining regions (HSRs) and less frequently as double minutes (DMs). In PCa, satDNA-AMPs have only been seen as HSRs, probably due to the small number of cases studied and reported for this phenomenon to date.^1^

The expression of satellite DNA in the form of RNA has been reported not only in PCas^2^ but also in the breast carcinoma cell line HeLa,^3^ where the transcription of satellite DNA has also been shown to be essential for the maintenance of centromere structure and function. In addition, satellite RNA has been detected in human tissues under heat shock.^4–6^ The latter is of interest in that satellite RNA has been shown to be involved in the recruitment of RNA processing (i.e. splicing factors) during stress response.^5,7^ It has been repeatedly shown that cellular stress is an inherent feature of carcinogenesis.^8^

In addition, RNA expression of satellite DNA has been observed in prenatal development of insects^9,10^ and mammals.^10,11^ Interestingly, similarities between cancer and prenatal tissue, such as chromothripsis, chromosome instability, and/or expression of (proto)oncogenes in both cases,^12^ have been observed in recent decades. Therefore, the expression of satellite DNA as RNA known from embryonic development,^9–11^ which has now also been detected in tumors,^2^ is not too surprising. However, the fact that heterochromatic satDNA-AMP in advanced tumors appears to be realized by HSR and/or DM formation has been overlooked until recently.^1^

## Materials and Methods

In our previous work on 31 primary PCas,^1^ we found satDNA-AMPs of D1Z1, D2Z1, D3Z1, D4Z1, D15Z4, D20Z1, and DYZ1 in five cases (16%; see Table 1). In this study, we investigated whether these satDNA-AMPs are associated with nearby localized euchromatic DNA amplifications in the affected five advanced PCas. To this end, DNA was extracted from the formalin-fixed, paraffin-embedded (FFPE) blocks of these five samples and analyzed by 180k chromosome microarray (CMA) according to Aust et al.^13^ As the studied PCa samples were 1–2 cm^3^ 10 sections each, 10 µm were sufficient for DNA extraction. The oligo-array method applied here involves comparative hybridization of the tested tumor DNA and a reference genomic DNA (Human Female Genomic DNA; Promega, Mannheim, Germany) on a chip with 170,334 specific oligonucleotide sequences (Agilent Human Genome CGH Microarray 180K; Agilent, Waldbronn, Germany). As analysis software, ‘workbench7’ (CytoGenomics; Agilent, Waldbronn, Germany) was applied. The results were analyzed according to the oligonucleotide position provided by Agilent in the UCSC Genome Browser on Human Feb. 2009 (GRCh37/hg 19) version.

**Table 1.** The Five PCas Studied Here, Their Gleason Scores, Their Satellite DNA Amplicons, and Regions With Copy Number Increases in Euchromatin.

Tumor Numbers According to Ariffen et al.^1^	Gleason Score	Satellite DNA Amplification as HSR	Gain of Copy Numbers Acc. to CMA
1	9	D20Z1	1q21.2q23.1 1q32.1q32.1 6p21.31p21.2 7q11.23q11.23 9q33q34.11 11q13.1q13.3 16pterp13.1
3	7	D15Z4	1q21.2q21.3 6p22.2p22.2
4	7	D3Z1, D4Z1	1q21.2q32.1 3p14.1p11.1 3q13.32q26.2 9q34.3qter 14q24.2q24.3 20p12.1p12.1
11	8	D2Z1	16q22.1q22.1 Xpterp22.33 Ypterp11.31
13	7	D1Z1, DYZ1	1q21.2q41 1q43qter 6p11.2p11.2 7q11.23q11.23 8q13.3q24.21 17q25.3qter

## Results

**Figure 1.**
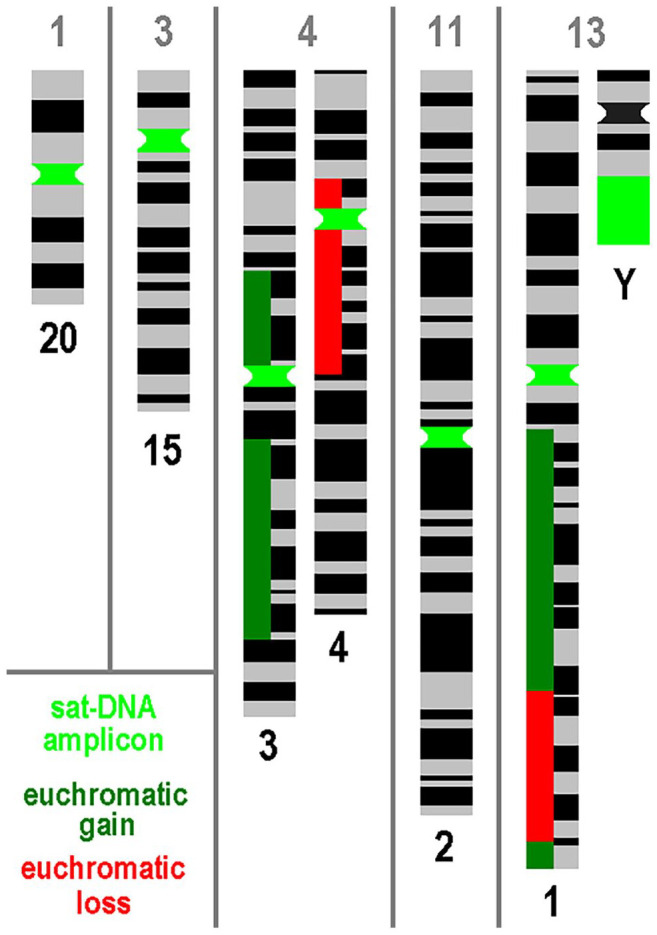
For the five PCas studied here (Nos. 1, 3, 4, 11, and 13), the affected chromosomes are shown with amplicons of satellite DNA (satDNA) together with the euchromatic gains and losses of these chromosomes according to Table 1 and Appendix Table 1. Abbreviations: DNA, deoxyribonucleic acid; PCa, prostate cancer.

The results are shown in Table 1 and summarized in Fig. 1: No nearby euchromatic copy number alterations (CNAs) were detected in the majority of chromosomes affected by satDNA-AMP as HSR (see also Appendix Table 1). This is true for cases 1, 3, 11, and 13 for both affected chromosomes, although chromosome 1 shows gains and losses along its long arm (Fig. 1). In case 4, satDNA-AMPs were found originating from the chromosomes 3 and 4: Whereas chromosome 3 shows a centromere-near gain in copy number for the short arm, there was a pericentric deletion of euchromatin in chromosome 4. In none of the cases was oncogene amplification detected by CMA.

In contrast, there were euchromatic centromere-near deletions in case 3 for chromosome 6p, case 4 for Yp and Yq, and euchromatic centromere-near gains in case 3 for chromosome 6p. All these euchromatic CNAs did not trigger satDNA-AMP in these chromosomes.

In total, the following euchromatic CNAs were found as gains two or more times in the five samples analyzed: 1q21q23.1~41, 6p22~21.31p21.1, and 7q11.23q11.23 and as losses: Xp22.32p22.31~11.3 and 8pterp22~11.2.

## Discussion

In this study, satDNA-AMPs were not associated with nearby localized euchromatic DNA amplification in any of the five cases examined. Overall, the euchromatic CNAs detected were as expected for PCas.^14,15^ Only one affected chromosome 4, each showed a moderate gain or loss of centromeric euchromatin (see Fig. 1). Overall, this seems to indicate that satDNA-AMPs may occur largely independently of euchromatic CNAs in advanced tumors. If this is true, the rarely observed coamplification of centromere-related oncogenes, such as *MDM2* and D12Z3 amplicons in lipomas, would be the exception rather than the rule for this type of amplicon formation. Should future studies identify satDNA-AMPs as DMs and HSR simultaneously in individual advanced tumors, satDNA-AMP, and amplification of oncogenes^16^ would be closely related mechanisms.

It should be noted that satDNA-AMP is not yet routinely accessed or accessible. Satellite DNAs can only be quantified by molecular cytogenetics.^17^ CMA, Sanger, and routine Next Generation Sequencing are blind to these DNA segments. This is, as these approaches do not cover heterochromatic regions and/or are not able to align or quantify highly repetitive sequences.^17^ Similarly, Ljubić et al.^2^ were only able to detect relatively small portions of alpha satellite DNA and not the centromeric alpha, beta, or satellite III DNAs, which are present as higher order repeat units, as studied by Ariffen et al.^1^

Of course, the present study has several limitations: (1) It is a spadework study performed in only five cases. In addition, (2) it is known that DNA extracted from FFPE material is partially degraded and results may be less accurate than with cryofixed or fresh postoperative tumor material.^18^ Still, as fluorescence in situ hybridization (FISH) and CMA studies on the here studied five FFPE-samples gave informative and easily evaluable results, DNA degradation did not seem to be an issue here. Furthermore, (3) tumor-specific euchromatic CNAs may not be complexly accessible as the tumor may be “contaminated” with normal cells.^19^ Although (1) and (2) can only be circumvented in future studies, for (3) it can be said that comingling with normal cells was excluded by evaluation of the used sections by two experienced pathologists (E.J. and B.T.); the tumor percentage was between 75% and 100%. In addition, the Phosphatase and TENsin homolog *(PTEN)* gene deletion previously found in case 13 by FISH was also detected in the CMA analyses, and no such deletions were detected in cases 1, 3, and 4 by either FISH or CMA. Only in case 11 there was a discrepancy, as the *PTEN* deletion was found in ~25% of the cells by FISH, which was not visible in CMA (see Appendix Table 1); this can be explained by an insufficient proportion of cells with CNAs.^19^

The findings that satDNA-AMP is not only present in advanced PCa samples^1^ but satDNA is also expressed in such samples suggest that both events are correlated with each other. Additional evidence that satDNA-AMP is a feature of advanced tumors is provided by its presence in human^17^ and murine-advanced tumor cell lines.^20^ Thus, it can be cautiously concluded that satDNA-AMP could be a new and independent marker for advanced tumor progression.

In case satDNA-AMP is suited as a marker for advanced tumors, it is possible that not only the presence of satDNA-AMP, but also multiple centromeres due to (partially) polyploid tumors could trigger tumor malignancy via the expression of satDNA. In addition, chromosomal heteromorphisms that cause an excessive expansion of satellite DNA should be investigated to determine whether they predispose to a more severe cancer progression. Further studies in other tumor types should be done to check if satDNA-AMP and satDNA-RNA-overexpression are present there as well in advanced samples.

## Appendix

**Appendix Table 1.** Complete Data of CMA Analyses of the Five Presented Cases.

Case 1		
Cytoband	Region Affected [GRCh37]	Kind of CNA
1q21.2q23.1	chr1:150257376-156860275	Gain
1q32.1q32.1	chr1:201732114-203428584	Gain
5q34q35.1	chr5:162956347-170262735	Loss
6p21.31p21.2	chr6:35381089-39207142	Gain
7q11.23q11.23	chr7:72365903-77469625	Gain
8pter-p22	chr8:193250-15186829	Loss
9q33q34.11	chr9:130052727-133273220	Gain
11q13.1q13.3	chr11:64543890-68785854	Gain
12q24.31qter	chr12:125740414-132321220	Loss
16pterp13.1	chr16:333753-5359467	Gain
Case 3		
Cytoband	Region Affected [GRCh37]	Kind of CNA
1q21.2q21.3	chr1:150257376-151669638	Gain
6p22.2p22.2	chr6:25947549-26285413	Gain
6p11.2p11.2	chr6:57246930-57489926	Loss
Xp22.32p22.31	chrX:4886357-8422893	Loss
Case 4		
Cytoband	Region Affected [GRCh37]	Kind of CNA
1p36.21p36.13	chr1:14336693-19510620	Loss
1q21.2q32.1	chr1:149103194-203445250	Gain
2q31.1q31.3	chr2:177202281-182606581	Loss
3p14.1p11.1	chr3:69436836-88183074	Gain
3q13.32q26.2	chr3:118575163-167925874	Gain
4p13p12	chr4:42495208-46580752	Loss
4q11q22.3	chr4:52689101-95362945	Loss
6p11.2p11.2	chr6:57270000-57503312	Gain
6q14q14	chr6:78979172-79023328	Loss
8pterp12	chr8:161472-33254022	Loss
9pterp22.2	chr9:204193-17697922	Loss
9q34.3qter	chr9:139722914-140240476	Gain
10q24.32qter	chr10:104490230-105279183	Loss
14q22.3q23.1	chr14:56058978-58667420	Loss
14q23.3q24.1	chr14:65795231-70110204	Loss
14q24.2q24.3	chr14:73566341-74384090	Gain
14q24.3q31.1	chr14:75412267-79535599	Loss
14q32.12q32.13	chr14:92421603-96078236	Loss
17p13.2p13.1	chr17:3903561-7744103	Loss
20p12.1p12.1	chr20:13370091-14315907	Gain
21q22.3qter	chr21:43097982-45659989	Loss
Ypterp11.2	chrY:2537972-10012121	Loss
Yq11q11.222	chrY:13213476-21098877	Loss
Case 11		
Cytoband	Region Affected [GRCh37]	Kind of CNA
1p36.32p36.23	chr1:2575457-8811612	Loss
16q22.1q22.1	chr16:66955572-70442715	Gain
16q23.3q23.3	chr16:82006620-83778055	Loss
21q22.2qter	chr21:40158830-42911329	Loss
Xpterp22.33	chrX:174905-2635590	Gain
Xp22.32p11.3	chrX:4886357-8422893	Loss
Ypterp11.31	chrY:124905-2585590	Gain
Case 13		
Cytoband	Region Affected [GRCh37]	Kind of CNA
1q21.2q41	chr1:149378207-216090561	Gain
1q41q43	chr1:216179405-241432469	Loss
1q43qter	chr1:242023918-249172869	Gain
6q14.1q16.2	chr6:80113727-99769419	Loss
7p14.1p12.1	chr7:40295934-53991940	Loss
7q11.23q11.23	chr7:73100444-76734076	Gain
8p23.3p12	chr8:193250-32336307	Loss
8q13.3q24.21	chr8:71086949-119886848	Gain
8q24.23q24.3	chr8:137364984-143388158	Loss
10q23.1q24.31	chr10:82027464-101901252	Loss
11q14.1qter	chr11:78541181-134828734	Loss
12q12q12	chr12:39213726-42672315	Loss
12q15q24.31	chr12:68980497-125740473	Gain
13q13q31	hr13:35831261-86543280	Loss
17q25.3qter	chr17:78787120-80993001	Gain
Xp22.33p22.31	chrX:4291483-8448318	Loss

Abbreviation: CNA, copy number alteration.

## References

[1] Ariffen NA Ornellas AA Alves G Shana’ah AM Sharma S Kankel S Jamali E Theis B Liehr T. Amplification of different satellite-DNAs in prostate cancer. Pathol Res Pract. 2024;256:155269. 38522124 10.1016/j.prp.2024.155269

[2] Ljubić S Sermek A Prgomet Sečan A Prpić M Jakšić B Murgić J Fröbe A Ugarković Đ Feliciello I. Alpha satellite RNA levels are upregulated in the blood of patients with metastatic castration-resistant prostate cancer. Genes. 2022;13:383. 35205427 10.3390/genes13020383 PMC8871578

[3] Quénet D Dalal Y. A long non-coding RNA is required for targeting centromeric protein A to the human centromere. eLife. 2014;3:e03254. 10.7554/eLife.03254 PMC4145801 25117489

[4] Eymery A Souchier C Vourc’h C Jolly C. Heat shock factor 1 binds to and transcribes satellite II and III sequences at several pericentromeric regions in heat-shocked cells. Exp Cell Res. 2010;316:1845–55. 10.1016/j.yexcr.2010.02.002 20152833

[bibr5-00221554251323657] Metz A Soret J Vourc’h C Tazi J Jolly C. A key role for stress-induced satellite III transcripts in the relocalization of splicing factors into nuclear stress granules. J Cell Sci. 2004;117:4551–8. 10.1242/jcs.01329 15331664

[6] Rizzi N Denegri M Chiodi I Corioni M Valgardsdottir R Cobianchi F Riva S Biamonti G. Transcriptional activation of a constitutive heterochromatic domain of the human genome in response to heat shock. Mol Biol Cell. 2004;15(2):543–51. 10.1091/mbc.E03-07-0487 PMC329232 14617804

[7] Chen LL Carmichael GG. Long noncoding RNAs in mammalian cells: what, where, and why? Wiley Interdiscip Rev RNA. 2010;1(1):2–21. 21956903 10.1002/wrna.5

[8] Lempesis IG Georgakopoulou VE Papalexis P Chrousos GP Spandidos DA. Role of stress in the pathogenesis of cancer (review). Int J Oncol. 2023;63(5):124. 37711028 10.3892/ijo.2023.5572 PMC10552722

[9] Sermek A Feliciello I Ugarković Đ. Distinct regulation of the expression of satellite DNAs in the beetle Tribolium castaneum. Int J Mol Sci. 2021;22:96. doi:10.3390/ijms22010296 PMC7796160 33396654

[bibr10-00221554251323657] Hall LE Mitchell SE O’Neill RJ. Pericentric and centromeric transcription: a perfect balance required. Chromosome Res. 2012;20(5):535–46. 10.1007/s10577-012-9297-9 22760449

[11] Thakur J Packiaraj J Henikoff S. Sequence, chromatin and evolution of satellite DNA. Int J Mol Sci. 2021;22:4309. 33919233 10.3390/ijms22094309 PMC8122249

[12] Cortés-Ciriano I Lee JJ Xi R Jain D Jung YL Yang L Gordenin D Klimczak LJ Zhang CZ Pellman DS ; PCAWG Structural Variation Working Group, Park PJ; PCAWG Consortium. Comprehensive analysis of chromothripsis in 2,658 human cancers using whole-genome sequencing. Nat Genet. 2020;52:331–41. 10.1038/s41588-019-0576-7 PMC7058534 32025003

[13] Aust N Schüle S Altendorf-Hofmann AK Chen Y Knösel T Dirsch O Settmacher U Weise A Mrasek K Liehr T. Loss of chromosome 4 correlates with better long-term survival and lower relapse rate after R0-resection of colorectal liver metastases. J Cancer Res Clin Oncol. 2013;139(11):1861–7. 10.1007/s00432-013-1505-2 PMC11824614 24061341

[14] Ishkanian AS Zafarana G Thoms J Bristow RG. Array CGH as a potential predictor of radiocurability in intermediate risk prostate cancer. Acta Oncol. 2010;49(7):888–94. 10.3109/0284186X.2010.499371 20590366

[15] Visakorpi T Kallioniemi AH Syvänen AC Hyytinen ER Karhu R Tammela T Isola JJ Kallioniemi OP. Genetic changes in primary and recurrent prostate cancer by comparative genomic hybridization. Cancer Res. 1995;55:342–7. 7529134

[16] Shimizu N. Gene amplification and the extrachromosomal circular DNA. Genes. 2021;12:1533. 34680928 10.3390/genes12101533 PMC8535887

[17] Liehr T. Chromosomal heteromorphisms and cancer susceptibility revisited. Cells. 2022;11:3239. 36291106 10.3390/cells11203239 PMC9600968

[18] Craig JM Vena N Ramkissoon S Idbaih A Fouse SD Ozek M Sav A Hill DA Margraf LR Eberhart CG Kieran MW Norden AD Wen PY Loda M Santagata S Ligon KL Ligon AH. DNA fragmentation simulation method (FSM) and fragment size matching improve aCGH performance of FFPE tissues. PLoS One. 2012;7(6):e38881. 10.1371/journal.pone.0038881 PMC3376148 22719973

[19] van de Wiel MA Picard F van Wieringen WN Ylstra B. Preprocessing and downstream analysis of microarray DNA copy number profiles. Brief Bioinform. 2011;12(1):10–21. 20172948 10.1093/bib/bbq004

[20] Hergenhahn L Padutsch N Azawi S Weiskirchen R Liehr T Rinčic M. Cytogenomic characterization of murine neuroblastoma cell line Neuro-2a and its two derivatives Neuro-2a TR-alpha and Neuro-2a TR-beta. Cells. 2024;13:1889. 39594637 10.3390/cells13221889 PMC11593031

